# A Community-Based Comprehensive Intervention Program for 7200 Patients with Type 2 Diabetes Mellitus in Chongqing (China)

**DOI:** 10.3390/ijerph111111450

**Published:** 2014-11-06

**Authors:** Li Qi, Liangui Feng, Wenge Tang, Xiangyu Ma, Xianbin Ding, Deqiang Mao, Jingxin Li, Yulin Wang, Hongyan Xiong

**Affiliations:** 1 Department of Epidemiology, College of Prevention Medicine, the 3rd Military Medical University, Chongqing 400038, China; E-Mails: qili19812012@126.com (L.Q.); xiangyuma1981@163.com (X.M.); 2 Chongqing Municipal Center for Disease Control and Prevention, Chongqing 400042, China; E-Mails: flg@cqcdc.org (L.F.); twg@cqcdc.org (W.T.); dingxianbing1970@163.com (X.D.); molly19812010@163.com (D.M.); wyl@cqcdc.org (Y.W.); 3 Jiangshu Municipal Center for Disease Control and Prevention, Jiangshu 210009, China; E-Mail: jingxinli2010@163.com; 4 The Center for Clinic Trials, Southwest Hospital, the 3rd Military Medical University, Chongqing 400038, China

**Keywords:** type 2 diabetes mellitus, intervention, community

## Abstract

This study assessed the feasibility of community-based comprehensive intervention on Type 2 diabetes mellitus (T2DM) on a large population in China. An intervention study was conducted on 7200 T2DM patients within one year and consisted of six lectures on health issues, and four times face-to-face lifestyle counseling delivered by general health practitioners, at local primary health centers (PHCs). A “knowledge, attitude and practice” (KAP) survey and fasting plasma glucose (FPG) measurement were conducted at baseline and after the intervention, respectively. A total of 6586 T2DM patients completed the intervention. After one year intervention, patients’ KAP level improved significantly (*p* < 0.001) and the average FPG has decreased from 8.53 mmol/L (standard deviation: 2.84) to 7.11 mmol/L (standard deviation: 1.34) (*p* < 0.001). Patients in rural areas and with lower education level showed higher FPG and poorer KAP level both before and after the intervention. In conclusion, community-based comprehensive intervention for T2DM is feasible on a large population. Improving and repeating the comprehensive strategy is greatly recommended in order to sustain the impact, especially in rural areas and for patients with lower education levels.

## 1. Introduction

Diabetes is a major public health problem worldwide because of its high prevalence, significant mortality and great public health burden [1,2,3]. A majority of the people with diabetes live in low- and middle-income countries. China is one of the developing countries where the prevalence of diabetes has increased rapidly over the last 30 years [4]. Data from five national surveys indicate that the prevalence of diabetes grew nearly 17-fold from 0.7% in 1980 [5] to 11.6% in 2010 [6]. Overall, more than 113 million Chinese adults are affected by diabetes at present [6], making China the leading nation globally in terms of the number of diabetes patients.

There is an urgent need to implement effective intervention strategies in China, particularly for type 2 diabetes (T2DM), which accounts for 90%–95% of all diagnosed cases of diabetes. In addition to medication (oral hypoglycemic agents and insulin), a large number of studies have indicated that non-pharmacological intervention on T2DM can play an effective role in prevention and control, involving lifestyle modifications such as improving diet, increasing physical activity, self-monitoring of health status as well as adherence to medication regimens [7,8,9]. Although some evidence demonstrated the efficacy of a comprehensive intervention (lifestyle modification along with pharmacological treatment) [10,11,12], most were implemented in resource-intensive settings with small sample sizes. Whether these strategies can be successfully implemented on a large population remains unclear, especially in economically disadvantaged regions.

At present, therapeutic lifestyle changes along with pharmacological approach are recommended by health associations like the American Diabetes Association [13], American Heart Association [14], and Chinese Diabetes Society [15]. According to the China Type 2 Diabetes Guidelines (2010) [15], health education activities, including regular health lectures and individual consultations are recommended at PHCs. Our aim was to evaluate the efficacy and feasibility of community-based intervention on a large-scale T2DM population in Chongqing, located in southwest China.

## 2. Materials and Methods

### 2.1. Design and Participants

We conducted a self-control study on 7200 patients with T2DM in Chongqing, located in southwest China. A multi-stage sampling method was used: stage 1, nine regions were randomly selected from all the 39 regions; stage 2, eight PHCs was randomly selected from each region. Finally, a total of 72 PHCs were selected. Then, recruitment information was posted to local communities in the respective areas of the selected 72 PHCs to recruit patients with T2DM in the period between September 2012 and December 2012. A total of 100 patients with T2DM who met our inclusion and exclusive criteria were registered for our study in each sampled PHC.

The inclusion criterion required that patients had to have been diagnosed with T2DM within the last six months prior to the study. We used the 1999 World Health Organization diagnostic criteria [16], in which T2DM was defined as a state at which fasting glucose level reaches 126 mg/dL (7.0 mmol/L), or 2 h postprandial glucose reaches 200 mg/dL (11.1 mmol/L), or both. Patients are excluded if they met any of the following criteria: uncontrolled complications (e.g., blindness or renal failure or previous amputation), mentally or physically handicapped, or participated in another lifestyle intervention programs. The study was approved by the Ethical Committee of Research in Chongqing Center for Disease Control and Prevention. Written informed consent was obtained from each participant.

### 2.2. Intervention

A total of 216 general practitioners (three persons per PHC) underwent a three-day intensive training to familiarize them with T2DM problems, before carrying out the intervention. Materials containing information about T2DM and China Type 2 Diabetes Guideline (2010) were prepared for every general practitioner. We conducted general intervention and individual intensive intervention for 1 year, between January 2013 and December 2013.

General intervention: All the patients were invited to attend six health lectures on diabetes holding every two months and lasting 1.5 h per session, conducted by trained general practitioners.

Individual intensive intervention: Besides six health care lectures, patients were accorded face to face individual counseling by general practitioners, every three months, lasting 15 to 20 min every time. The general practitioner assessed patients’ conditions and put them on a proper diet, physical activity and pharmacological treatment advice, according to the China Type 2 Diabetes Guidelines (2010). Table 1 presents key aspects of the intervention.

**Table 1:** The community-based comprehensive intervention.

Items		Core Contents	
General intervention	Six health lectures, held bimonthly, lasting 1.5 h every time, led by trained general practitioners	Session 1	Basic knowledge of diabetes
		Session 2	Risk factors for T2DM
		Session 3	Nutrition and physical activity in T2DM management
		Session 4	How to prevent diabetes-related complications
		Session 5	Management diabetes for life, as foot-care, stress management, fatigue management and usage of insulin
		Session 6	Monitoring diabetes and seeking support from family and friends
Intensive intervention	Individual consultation delivered by general practitioners face to face, every three months, lasting 15 to 20 minutes every time	Pharmacological treatment	Based on China Type 2 Diabetes Guideline (2010) Tips-Emphasize the importance of taking oral medication or insulin in accordance with general practitioner’s prescription.
		Increased physical activity	Goal-At least 150 minutes per week of moderate-intensity aerobic exercise (equivalent to walking). Tips-Engage in physical activity during leisure time and commuting as much as possible; Provide participant with pedometer and encourage them to record the minutes every day. Information leaflets about exercise education were given to all participants.
		Dietary modification	Goal-Low in saturated fats (less than 30% of the total fats), increased portions of fiber and vegetables, and maintained an appropriate total calorie intake goal of 1200–1800 kcal per day. Tips-Intake appropriate amounts of fish, eggs, low-fat milk, lean meat; Reduction simple sugars and refined carbohydrates; Increase fiber-rich food, such as whole grains, brown rice, vegetables and fruits. Record food types and portion sizes for one day every three days. Written nutrition education materials were given to all participants.
		Weight reduction	Goal-Reduce BMI (body mass index categories) to <24 kg/m^2^.Tips-Participants were asked to weight themselves once a month. Encourage participants (overweight or obesity) to lose weight by decreasing energy intake and increasing physical activity.
		Others	Goals-Smoking cessation (if smoker) and limit alcohol intake (if drinking) to ≤ 2 drinks/day, including 1–2 alcohol-free days/week. Tips-General practitioners emphasized the dangers Of tobacco use and excessive drinking to blood glucose control and provided DVDs to patients.

Abbreviation: T2DM, type 2 diabetes mellitus; DVD, digital video disk

### 2.3. Evaluation

Participants were assessed for the condition of T2DM at baseline and after 1 year intervention under the same parameters. A suitably designed KAP questionnaire was administered face-to-face by trained interviewers. We designed the KAP questionnaire based on Diabetes Self-care Activities Questionnaire [17], International Physical Activity Questionnaire [18] and Chinese Food Frequency Questionnaire [19], and modified to improve the applicability in PHCs. The questionnaire was reviewed through focus group discussions and was pilot-tested for internal consistency and reliability beforehand in 50 patients with diabetes.

The questionnaire covered four areas: social-demographic characteristics, T2DM-related health knowledge, attitude and self-care practices towards T2DM:

(a) Awareness of T2DM-related knowledge was evaluated using seven questions relating to diagnostic criteria of hypertension and T2DM, typical symptoms, risk factors and main complications of T2DM, and then categorized as known or unknown.
(b) Attitudes towards T2DM, assessed using four questions relating to the cognitive rate of the importance of medication compliance, physical activity and dietary modification, and the positive attitudes towards having diabetes were surveyed and then categorized as “agree” or “disagree”.
(c) Self-care practices: Physical activity modification, dietary modification and medication compliance.

- Physical activity was assessed by Physical activity recall at the last seven days. Participants were classified as adherence or non-adherence which equates to distinguishing between those who achieved at least 150 minutes per week of moderate-intensity aerobic exercise (equivalent to walking) and those who do not [20].
- Dietary nutrient intake was assessed by 24 h recall of the types of food consumed. We calculated energy intake for individual food items with Dietary Guidelines for Chinese Residents [21]. Participants were classified as adherence and non-adherence, which equates to distinguishing between those who achieved the current dietary recommendations and those who do not [20].
- Medication compliance was assessed by use of medical records, and defined as “take oral medication” or “insulin” in accordance with general practitioner’s prescription.

In addition, fasting plasma glucose (FPG) measurement was completed by trained laboratory technicians and the overnight fasting venous specimen collected in the morning using a vacuum tube containing sodium fluoride for measuring FPG. All the measurements were performed at local PHCs laboratories using the unified hexokinase method (instrument model: TBA-40FR, Toshiba, Tokyo, Japan).

### 2.4 Statistical Methods

Initial data was entered into EpiData software 3.1 and analyzed using SPSS software18.0. Data from continuous variables were presented as mean ± standard deviation (SD) while categorical variables were presented as percentages. Pearson Chi-square and Paired-Samples *t*-test were used to examine pre-post changes of KAP levels and FPG. Student’s t- test and analysis of the variance (ANOVA) were used to examine the FPG between patients in different levels of urbanization (rural *vs.* urban) and education (≤ primary school, middle school and ≥ high school).

## 3. Results

### 3.1. Baseline Characteristics of the Participants

Of the 7200 T2DM patients recruited at baseline, 6586 (91.5%) completed the study, 31 (0.43%) had poor compliance and were referred to secondary or tertiary care centers, 22 (0.31%) moved to another place, 390 (5.42%) attended the health lectures less than six times while 171 (2.38%) failed to complete the evaluation post-intervention. Of the remaining 6586, 2503 (38.0%) were male and 4083 (62.0%) were female. Mean age ± SD was 64.14 ± 10.64. The characteristics of participants were shown according to the level of urbanization (Table 2).

**Table 2:** Characteristics of participants in our study.

Variables	Total	Urban	Rural
N	6586	2778	3808
Gender, n (%)			
Male	2503 (38.0%)	994 (35.8%)	1509 (39.6%)
Female	4083 (62.0%)	1784 (64.2%)	2299 (60.4%)
Age, n (%)			
≤49 years	718 (10.9%)	199 (7.2%)	519 (13.6%)
50–59 years	1294 (19.7%)	579 (20.8%)	715 (18.8%)
60–69 years	2495 (37.9%)	1040 (37.4%)	1455 (38.2%)
70–79 years	1683 (25.5%)	766 (27.6%)	917 (24.1%)
≥80 years	396 (6.0%)	194 (7.0%)	202 (5.3%)
Marriage Status, n (%)			
Single	98 (1.5%)	30 (1.1%)	68 (1.8%)
Married	5750 (87.3%)	2419 (87.1%)	3331 (87.5%)
Widowed	685 (10.4%)	291 (10.4%)	394 (10.3%)
Divorced	53 (0.8%)	38 (1.4%)	15 (0.4%)
Ethnics (Han), n (%)	6157 (93.5%)	2699 (97.2%)	3458 (90.8%)
Education level, n (%)			
≤Primary school	4185 (63.5%)	1374 (49.5%)	2811 (73.8%)
Middle school	1558 (23.7%)	834 (30.0%)	724 (19.0%)
≥High school	843 (12.8%)	570 (20.5%)	273 (7.2%)

### 3.2. Changes of Knowledge, Attitudes and Practices

Table 3 and Table 4 show participants’ changes of KAP level. Significant improvement was noted with regard to the awareness of T2DM-related health-knowledge in total and different levels of urbanization and education (*p* < 0.001). The cognitive rate of the importance of medication compliance, physical activity modification, dietary modification and self-monitoring of blood glucose significantly increased (from 73.55% to 94.15%, change: 20.60; 48.16% to 80.31%, change: 32.15% and 66.95% to 83.86%, change: 16.91%, respectively; *p* < 0.001). The percentage of participants who believed T2DM is preventable and controllable also significantly increased from 65.00% to 92.04% (*p* < 0.001).

Percentage of participants’ adherence to medication compliance, physical activity modification and dietary modification significantly increased (from 19.48% to 75.19%, change: 55.71%; 19.02% to 68.10%, change: 49.08%; 18.78% to 41.62%, change: 22.84%, respectively; *p* < 0.001).

**Table 3:** Changes of patients’ KAP pre- and post- intervention according to urbanization level.

Variables	Urban (n = 2778)				Rural (n = 3808)				Total (n = 6586)			
	Rate before (%)	Rate after (%)	x^2^	*p*	Rate before (%)	Rate after (%)	x^2^	*p*	Rate before (%)	Rate after (%)	x^2^	*p*
Knowledge												
Diagnostic criteria for HP	70.63	90.50	350.21	<0.001	65.39	80.46	219.11	<0.001	67.60	84.69	529.93	<0.001
Diagnostic criteria for T2DM	63.61	78.51	150.00	<0.001	50.18	84.77	1037.75	<0.001	55.85	82.13	1063.24	<0.001
Typical symptoms for T2DM	78.94	89.99	129.29	<0.001	68.43	82.12	191.50	<0.001	72.87	85.44	315.42	<0.001
Risk factors of T2DM												
Lack of physical exercise	82.04	90.19	73.08	<0.001	69.56	80.05	163.73	<0.001	74.83	84.33	232.99	<0.001
Unhealthy diet	76.96	87.33	101.79	<0.001	71.45	83.14	148.17	<0.001	73.78	84.91	248.87	<0.001
Smoking	61.45	76.24	141.75	<0.001	55.91	69.77	156.76	<0.001	58.24	72.50	295.71	<0.001
Main complications of T2DM	47.34	82.43	750.95	<0.001	34.56	65.76	698.24	<0.001	39.95	72.80	1445.59	<0.001
Attitude												
Importance of medication compliance	83.05	96.08	252.27	<0.001	66.62	92.75	803.09	<0.001	73.55	94.15	1032.47	<0.001
Importance of physical activity modification	51.66	87.51	843.59	<0.001	45.59	75.05	690.60	<0.001	48.16	80.31	1481.01	<0.001
Importance of dietary modification	72.61	86.79	172.68	<0.001	62.82	81.72	339.64	<0.001	66.95	83.86	507.97	<0.001
T2DM is preventable	74.44	93.77	388.29	<0.001	58.11	90.78	1068.17	<0.001	65.00	92.04	1427.91	<0.001
Practice												
Medication compliance	15.55	80.92	2377.22	<0.001	22.35	71.01	1811.36	<0.001	19.48	75.19	4099.58	<0.001
Physical activity modification	19.69	69.69	1404.84	<0.001	18.54	66.94	2020.99	<0.001	19.02	68.10	3440.67	<0.001
Dietary modification	28.08	56.19	450.28	<0.001	12.00	30.99	406.75	<0.001	18.78	41.62	814.66	<0.001

Abbreviations: KAP, knowledge, attitude and practice; HP, hypertension; T2DM, type 2 diabetes mellitus.

**Table 4:** Changes of patients’ KAP pre- and post- intervention according to education level.

Variables	Primary School or Less (n = 4185)				Middle School (n = 1558)				High School or Above (n = 843)			
	Rate before (%)	Rate after (%)	x^2^	*p*	Rate before (%)	Rate after (%)	x^2^	*p*	Rate before (%)	Rate after (%)	x^2^	*p*
Knowledge												
Diagnostic criteria for HP	66.18	82.91	308.57	<0.001	70.86	85.76	101.68	<0.001	68.63	91.61	138.76	<0.001
Diagnostic criteria for T2DM	54.68	82.96	779.33	<0.001	57.76	82.42	225.73	<0.001	58.07	77.46	72.17	<0.001
Typical symptoms for T2DM	65.59	81.84	285.88	<0.001	86.33	89.67	8.22	<0.001	84.10	95.49	59.67	<0.001
Risk factors of T2DM												
Lack of physical exercise	71.70	83.61	171.40	<0.001	80.36	87.67	31.06	<0.001	80.12	90.41	35.66	<0.001
Unhealthy diet	71.74	84.11	186.34	<0.001	76.82	85.94	42.72	<0.001	78.26	86.95	22.01	<0.001
Smoking	57.88	72.64	201.26	<0.001	59.56	71.69	50.82	<0.001	57.65	73.31	45.72	<0.001
Main complications of T2DM	36.20	70.63	997.00	<0.001	44.35	74.71	298.03	<0.001	50.42	79.95	116.67	<0.001
Attitude												
Importance of medication compliance	71.16	93.73	735.37	<0.001	79.97	95.31	169.28	<0.001	73.55	94.15	132.72	<0.001
Importance of physical activity modification	41.89	77.82	977.24	<0.001	52.82	85.69	395.11	<0.001	70.46	82.68	35.08	<0.001
Importance of dietary modification	64.43	83.54	397.21	<0.001	71.82	84.79	77.08	<0.001	70.46	83.74	42.15	<0.001
T2DM is preventable	59.32	92.11	1224.74	<0.001	71.82	90.32	173.42	<0.001	80.59	94.90	80.63	<0.001
Practice												
Medication compliance	13.20	69.77	2759.88	<0.001	20.25	80.62	1136.77	<0.001	49.23	92.05	372.70	<0.001
Physical activity modification	15.87	61.51	1906.66	<0.001	20.20	75.42	1063.17	<0.001	32.50	87.31	678.30	<0.001
Dietary modification	6.18	33.60	986.37	<0.001	30.64	42.49	47.36	<0.001	59.43	79.83	82.98	<0.001

Abbreviations: KAP, knowledge, attitude and practice; HP, hypertension; T2DM, type 2 diabetes mellitus.

### 3.3. Changes of FPG

After one year intervention, the average FPG had decreased from 8.53 ± 2.84 mmol/L to 7.11 ± 1.34 mmol/L (*p* < 0.001). In urban areas, the average FPG decreased from 7.72 ± 2.52 mmol/L to 6.83 ± 1.19 mmol/L (*p* < 0.001), while it decreased from 9.11 ± 2.92 mmol/L to 7.31 ± 1.40 mmol/L (*p* < 0.001) in rural areas. Table 5 and Table 6 present the FPG decrease after 1-year follow-up; significant decrease was found in total and different level of urbanization and education (*p* < 0.001).

In addition, we compared the participants’ FPG levels across varying levels of urbanization and education both before and after intervention, results showed that FPG of participants in rural areas was significantly higher (t_before_ = 35.40, *p* < 0.001; t_after_ = 25.62, *p* < 0.001) than those in urban regions. Significant differences of FPG was also recorded among participants with different education levels both at the beginning and end of intervention (F_before_ =18.62, *p* < 0.001; F_after_ =36.40, *p* < 0.001).

**Table 5:** Changes of participants’ FPG according to urbanization level (Mean ± SD).

Area	N	Before (mol/L)	After 1-Year Intervention (mmol/L)	t	*p*
Urban	2778	7.72 ± 2.52	6.83 ± 1.19	20.66	<0.001
Rural	3808	9.11 ± 2.92	7.31 ± 1.40	41.05	<0.001
Total	6586	8.53 ± 2.84	7.11 ± 1.34	44.71	<0.001

Abbreviations: FPG, fasting plasma glucose; SD, standard deviation.

**Table 6:** Changes of participants’ FPG according to education level (Mean ± SD).

Education Level	N	Before (mmol/L)	After 1-Year Intervention (mmol/L)	t	*p*
≤ Primary school	4185	8.82± 2.91	7.19 ± 1.39	39.41	<0.001
Middle school	1558	8.19 ± 2.74	7.03 ± 1.26	19.05	<0.001
≥ High school	843	7.68 ± 2.39	6.87 ± 1.18	11.03	<0.001
Total	6586	8.53 ± 2.84	7.11 ± 1.34	44.71	<0.001

Abbreviations: FPG, fasting plasma glucose; SD, standard deviation.

## 4. Discussion

On a large population, our study indicated that a comprehensive intervention strategy was effective on decreasing T2DM patient’s FPG from 8.53 mmol/L (SD: 2.84) to 7.11 mmol/L (SD: 1.34) for 1-year. Our results were similar to the report by Kim (FPG reduction: 1.6 mmol/L) [22], but lower recorded figures than the trial by Mitra (FPG reduction: 2.67 mmol/L) [23].

It is worth noting that patients in rural areas and with lower education level showed higher FPG, both before and after intervention. The allocation of medical health resources is uneven in China with investments in rural health being far below the urban level. A lower educational level has been associated with poorer self-care ability and might influence the glucose control [24]. Thus, it is advisable to pay more attention to T2DM prevention and control in rural areas and patients with lower education level in China.

Previous studies have consistently shown that patients with good knowledge about diabetes have better long term glucose control [24,25,26]. Thus, it is vital to ensure that patients’ knowledge is adequate and their attitudes and practices right. In our study, the awareness of type 2 diabetes-related health-knowledge was very poor at baseline, especially for patients in rural areas and with lower education level which was in accordance with previous studies conducted in both developed and developing countries [27,28]. After 1-year intervention, the overall awareness improved significantly, especially on awareness rate of major complications of T2DM, which may be an important factor influencing glucose level. However, the proportion of patients who realized the main complications of T2DM and the health hazards of smoking remain inadequate (72.80% and 72.50%). Patients’ ignorance of the serious complications of T2DM might cause reluctance to change unhealthy lifestyles that would affect the severity of diabetes. Smoking is strongly linked to the risk of diabetes morbidity as well as mortality [29,30]. China has the highest tobacco consumption rate worldwide [31,32], therefore, effective interventions to support smoking cessation are urgently needed.

In terms of the attitudes and practices towards diabetes self-care, our study showed great improvement after 1 year’s intervention, especially on the cognitive rate of the importance of medication compliance and participants’ adherence to medication compliance. This result indicated that medication compliance may be a significant influence factor on glucose level. It is worth noting that participants’ attitudes and practices were not consistent with each other as it proved that the participants’ practices were poorer than attitudes. Although 94.15%, 80.31% and 83.86% patients realized the importance of medication compliance, increased physical activity and dietary modification after one year intervention, only 75.19%, 68.10% and 41.62% respectively had adhered to it. The currently applied intervention focused on health education and might be lacking strict encouraging mechanism; therefore, it faces challenges in putting knowledge into practice. Thus, improving intervention strategy is greatly recommended in the future. For example, we could establish encouraging mechanism by offering free physical examinations and health equipment such as salt spoon, oil pot and BMI ruler, or by use of innovative tools like “timers” to remind patients to take their medication or engage in daily physical activities, *etc.* In addition, increased intervention frequency through mobile health technology based on web [33] or mobile telephone [34,35,36] might be included in our future study.

To the best of our knowledge, this is the widest study on community-based comprehensive management strategy for T2DM in China, and it is the first time to evaluate the effectiveness of T2DM management strategy by the reduction of patients’ blood glucose and improvement of the KAP level at the same time. It is important to note that the strategy appears to be feasible for T2DM management in both rural and urban regions according to the practicability and high retention rate observed in our study. We expect more significant benefits after long-term intervention.

Our study has several limitations. First, the study aimed at examining the efficacy and feasibility of the community-based intervention on a large population. Enhancing medication compliance and lifestyle modification were both involved; therefore, it is difficult to determine which intervention played a major role. Second, the changes of patients’ practices on diabetes self-care were measured by self-report in questionnaires that might encounter some biased responses. Third, we measured FPG to examine the change of blood glucose level but did not measure glycosylated hemoglobin due to insufficient testing equipment in PHCs and limited funding. Further research involving more evaluating indicators is needed. Finally, we were unable to conduct a randomized controlled trial which is difficult to conduct considering that medication compliance education and lifestyle modification are recommended in all PHCs, in China. Regardless, any future study should use a randomized controlled trial to make a definitive conclusion about the efficacy of the intervention.

## 5. Conclusions

In conclusion, this study shows that community-based comprehensive intervention for T2DM is feasible and effective on a large population. The currently applied intervention might be lacking strict encouraging mechanisms and faces challenges in putting knowledge into practice. Improving intervention strategy is greatly recommended in the future, especially in rural areas and for patients with lower education levels.
